# Serum superoxide dismutase level is a potential biomarker of disease prognosis in patients with HEV-induced liver failure

**DOI:** 10.1186/s12876-022-02095-2

**Published:** 2022-01-09

**Authors:** Yajuan He, Fei Wang, Naijuan Yao, Yuchao Wu, Yingren Zhao, Zhen Tian

**Affiliations:** 1 Department of Ultrasound, The First Affiliated Hospital of Xi’an Jiaotong University, 277 West Yanta Road, Xi’an City, 710061 Shaanxi Province China; 2 Department of Infectious Diseases, The First Affiliated Hospital of Xi’an Jiaotong University, Xi’an City, Shaanxi Province China

**Keywords:** HEV, Liver failure, Oxidative stress, HMGB1, Apoptosis

## Abstract

**Background:**

Viral hepatitis E clinically ranges from self-limiting hepatitis to lethal liver failure. Oxidative stress has been shown to mediate hepatic inflammation during HBV-induced liver failure. We investigated whether a biomarker of oxidative stress may be helpful in assessing severity and disease outcomes of patients with HEV-induced liver failure.

**Methods:**

Clinical data were obtained from patients with HEV-induced acute viral hepatitis (AVH, n = 30), acute liver failure (ALF, n = 17), and acute-on-chronic liver failure (ACLF, n = 36), as well as from healthy controls (HC, n = 30). The SOD and HMGB1 levels were measured in serum by ELISA. HL-7702 cells were cultured and stimulated by serum from HEV-infected patients or by HMGB1; oxidative status was investigated by CellROX and apoptosis was investigated by flow cytometry.

**Results:**

Patients with HEV-induced liver failure (including ALF and ACLF) showed increased SOD levels compared with HEV-AVH patients and healthy controls. SOD levels > 400 U/mL were associated with a significantly higher risk of mortality in HEV-ALF and HEV-ACLF patients. Serum from HEV-infected patients led to ROS accumulation, HMGB1 secretion, and apoptosis in HL-7702 cells. Antioxidant treatment successfully inhibited HEV-induced HMGB1 secretion, and HMGB1 promoted apoptosis in HL-7702 cells.

**Conclusion:**

HEV increased oxidative stress in the pathogenesis of HEV-induced hepatic diseases. Early testing of serum SOD may serve as a predictor of both HEV-ALF and HEV-ACLF outcomes. Moreover, development of strategies for modulating oxidative stress might be a potential target for treating HEV-induced liver failure patients.

## Background

Globally, an estimated 2 billion people live in hepatitis E virus (HEV) endemic areas, of which majority are in developing countries, including China. Outcomes of HEV infection vary from asymptomatic acute viral hepatitis (AVH) to lethal acute liver failure (ALF). Recent studies have shown that an HEV infection can result in a severe disease in patients with chronic hepatitis B (CHB), and studies of serum epidemiology in China have shown a 17.6% superinfection rate of HEV in CHB patients [1]. Moreover, HEV infection has been shown to account for acute-on-chronic liver failure (ACLF) in patients with alcoholic liver disease (ALD) [2].

Liver failure is a rare but life-threatening critical disease, including ALF which occurs most often in patients without preexisting liver disease and ACLF which defines an acute decompensation in patients with chronic liver disease [3, 4]. Currently, there are few effective therapies apart from liver transplantation. Given that liver failure is a life-threatening disease with high mortality rate, scoring systems for assessing its severity and disease outcomes have been developed, including King’s College Criteria, the SOFA score, and the Model for End-Stage Liver Disease (MELD) score [4, 5]. However, all these methods focus on impaired liver functions, whereas few studies have concentrated on disease pathogenesis.

Liver failure is characterized by excessive hepatocyte death and liver inflammation, and patients with liver failure often present with endotoxemia and increased serum LPS levels due to increased gut permeability [6]. Recent studies have found that high-mobility group box 1 (HMGB1), a late mediator of endotoxic shock, serves as a prognostic biomarker indicating disease prognosis of ALF and ACLF patients, including HEV-induced ALF patients [7–9].

Oxidative stress is believed to play an important role in advanced liver failure. Previous studies have revealed that reactive oxygen species (ROS) play a role in hepatic inflammation [10], and ROS also promote HMGB1 secretion [11]. It has also been suggested that ROS in hepatocytes play important roles in the pathogenesis of liver failure; injured/dead hepatocytes greatly increase oxidative stress, which in turn contributes to further hepatocyte loss and impedes regeneration, culminating in a vicious cycle [12]. Since liver failure is a process with increased systemic oxidative stress mediated by ROS during disease pathogenesis, we intended to investigate whether a biomarker of oxidative stress may predict severity and prognosis of HEV-induced liver failure.

Superoxide dismutase (SOD) transforms toxic superoxide into hydrogen peroxide, thereby limiting the detrimental effects of ROS. Our previous study has shown an increased plasma SOD level in ALF patients, which may be an adaptive response to elevated oxidative stress; meanwhile, plasma SOD level was associated with disease severity in ALF patients [12]. SOD staining in ALF liver tissue revealed an increased expression of SOD2, also known as manganese-dependent SOD (MnSOD), which mainly eliminates mitochondrial ROS in cells and plays an antioxidant role [13]. Thus far, no studies have evaluated the severity and prognosis of HEV-induced liver failure from the perspective of hepatic oxidative stress. Therefore, the aim of the present study was to identify whether plasma SOD may be a predictive indicator in patients with HEV-induced liver failure.

## Materials and methods

### Patients

From January 2017 to December 2019, a total of 53 patients diagnosed with HEV induced liver failure, including 21 HEV-ACLF patients on the background of CHB, 15 HEV-ACLF patients on the background of ALD, and 17 HEV-ALF patients, were enrolled at the First Affiliated Hospital of Xi’an Jiaotong University, Shaanxi, China. All participants provided written informed consent, depending on the patient’s altered mental status, and the study was approved by the Research Ethics Committee of the First Affiliated Hospital of Xi’an Jiaotong University.

Patients were diagnosed with ACLF based on the following criteria of the Asian Pacific Association for the Study of the Liver (APASL): (1) serum bilirubin ≥ 85 mol/L; (2) international normalized ratio (INR) ≥ 1.5 or prothrombin activity ≤ 40%; (3) any degree of encephalopathy and/or clinical ascites within 4 weeks; (4) and an evidence of ongoing chronic liver disease.

Patients were diagnosed with ALF based on the following criteria of APASL: (1) coagulation abnormalities, typically with an INR ≥ 1.5 or prothrombin activity ≤ 40%; (2) any degree of encephalopathy; (3) no preexisting cirrhosis, and with an illness duration of < 26 weeks.

Patients diagnosed with ACLF or ALF were all aged from 18 to 75 years old.

A total of 66 patients in our cohort were excluded for the following reasons: (1) manifestation of decompensated liver cirrhosis prior to liver failure diagnosis, such as ascites and variceal hemorrhage; (2) trans-jugular intrahepatic portosystemic shunt (TIPS) in patients with portal hypertension; (3) pathological diagnosis or clinical susception of hepatocellular carcinoma; (4) other malignancies such as gastric cancer; (5) pregnancy; (6) HIV or hepatotropic virus infection.

We calculated the MELD score using the standard formula: 11.2 × ln (INR) + 9.57 × ln (creatinine, mg/dL) + 3.78 × ln (bilirubin, mg/dL), with a lower limit of 1 for all variables.

During the same period, age- and sex-matched 30 healthy participants and 30 HEV-AVH patients were recruited as controls.

### Detections

Serum SOD levels were measured using an ELISA commercial kit (#EIASODC, Thermo Fisher Scientific, Waltham, MA, USA) in according with the manufacturer’s protocol at hospital administration. Samples and standards were run in duplicate, and the sensitivity of the assay was 0.044 U/mL.

HMGB1 was measured by a commercially available ELISA kit (Cusabiotech, Hubei, China). This assay recognizes recombinant as well as natural human HMGB1 without significant cross-reactivity or interference.

The diagnosis of acute HEV infection was based on the detection of HEV-RNA by polymerase chain reaction (PCR) and anti-HEV IgM with a commercial kit (Genelabs Technologies, Singapore). All of the recruited subjects had the typical profile of positive HEV-RNA and anti‐HEV IgM before the onset of liver failure. HBsAg, HBsAb, HBeAg, HBeAb, and HBcAb were detected with an automatic rapid immunoassay system (AxSYM, Abbott, USA).

### Cell culture

HL-7702 cells were cultured in DMEM medium supplemented with 10% fetal bovine plasma and 2 Mm L-glutamine at 37 °C in a 95% air, 5% CO_2_-humidified atmosphere. Cells were trypsinized, and 5 × 10^5^ cells were seeded onto plastic dishes and then treated with serum from HEV-AVH, HEV-ALF and HEV-ACLF patients, and 10 mM N-Acetyl-L-cysteine (NAC).

### Quantitation of apoptosis

To quantify apoptosis, flow cytometry was used. A total of 2.0 × 10^4^ HL-7702 cells were seeded into each well of a 12-well plate. The following day, the cells were washed with a new medium and exposed to various reagents, as indicated. At harvest, the cells were washed and harvested with PBS and fixed with 3.7% paraformaldehyde in PBS for 10 min at room temperature. Annexin V-PI double-staining was performed in according with the manufacturer’s instructions (#640932, BD Biosciences, Franklin Lakes, NJ, USA). Briefly, 5 μL of Annexin V conjugate and PI were added to 100 μL of cell suspension for 15 min, and then, 400 μL of binding buffer was added and mixed gently while the samples were kept on ice. Flow cytometric analyses were performed with BD Jazz (BD FACS Jazz).

### Statistical methods

Results are presented as means and standard deviations (SDs). Demographics were compared for categorical variables using a chi-squared test or Fisher’s exact test as appropriate, and for continuous variables using a Wilcoxon rank sum test. ROC curves were generated using logistic regression where the event was death within 90 days post-enrollment. Cut-offs for continuous variables were determined as the maximum of the sum of sensitivity and specificity. Kaplan–Meier survival curves to 90 days post-admission were compared using log-rank tests. Data were analyzed using SPSS version 16.0 software (IBM Corporation, Somers, NY, USA). Differences were considered to be of statistical significance when the *P* value < 0.05.

## Results

### Baseline characteristics

The current study cohort was composed of a population with admission criteria including coagulopathy and encephalopathy as described above. The clinical baseline characteristics of HEV-induced liver failure patients, HEV-AVH patients and healthy controls in the present study are shown in Table 1.

**Table 1:** Demographic data and clinical characteristics

Parameter	HC (n = 30)	HEV-AVH (n = 30)	HEV-ALF (n = 17)	HEV-ACLF (n = 36)
Age (yr)	41.34 ± 7.55	44.43 ± 4.38	46.43 ± 8.34	48.88 ± 10.67
Gender(M/F)	23/7	23/7	13/4	27/9
PTA (%)	83.54 ± 15.77	77.23 ± 16.87	42.33 ± 13.22	38.02 ± 14.34
FIB (g/L)	2.28 ± 0.67	2.13 ± 0.95	1.56 ± 0.54	1.43 ± 0.56
INR	1.31 ± 0.29	1.35 ± 0.34	2.01 ± 0.34	2.17 ± 0.45
WBC(1 × 10^9^/L)	5.43 ± 1.24	5.21 ± 1.34	15.34 ± 6.67	16.28 ± 8.54
PLT (1 × 10^9^/L)	223.47 ± 34.34	187.04 ± 56.21	123.65 ± 23.45	112.77 ± 62.43
ALT (U/L)	25.23 ± 12.34	25.45 ± 11.32	376.56 ± 399.23	389.52 ± 413.93
GLU (mM)	4.49 ± 0.65	4.65 ± 0.89	5.54 ± 2.23	5.74 ± 3.36
TBIL (μM)	14.33 ± 3.75	17.88 ± 7.98	296.66 ± 154.23	323.61 ± 134.52
CHOL (mM)	4.01 ± 0.39	3.25 ± 0.78	2.65 ± 18.56	2.54 ± 0.81
CREA (μM)	46.66 ± 11.54	55.61 ± 12.01	59.34 ± 15.76	63.54 ± 20.75
MELD			24.99 ± 4.23	25.15 ± 4.67

ALT, alanine aminotransferase; CHOL, cholesterol; CREA, creatinine; FIB, fibrinogen; GLU, glucose; INR, international normalized ratio; PLT, platelet count; PTA, prothrombin activity; TBIL, total bilirubin; WBC, white blood cell count

### HEV-liver failure patients show increased mortality rate

**Fig. 1:**
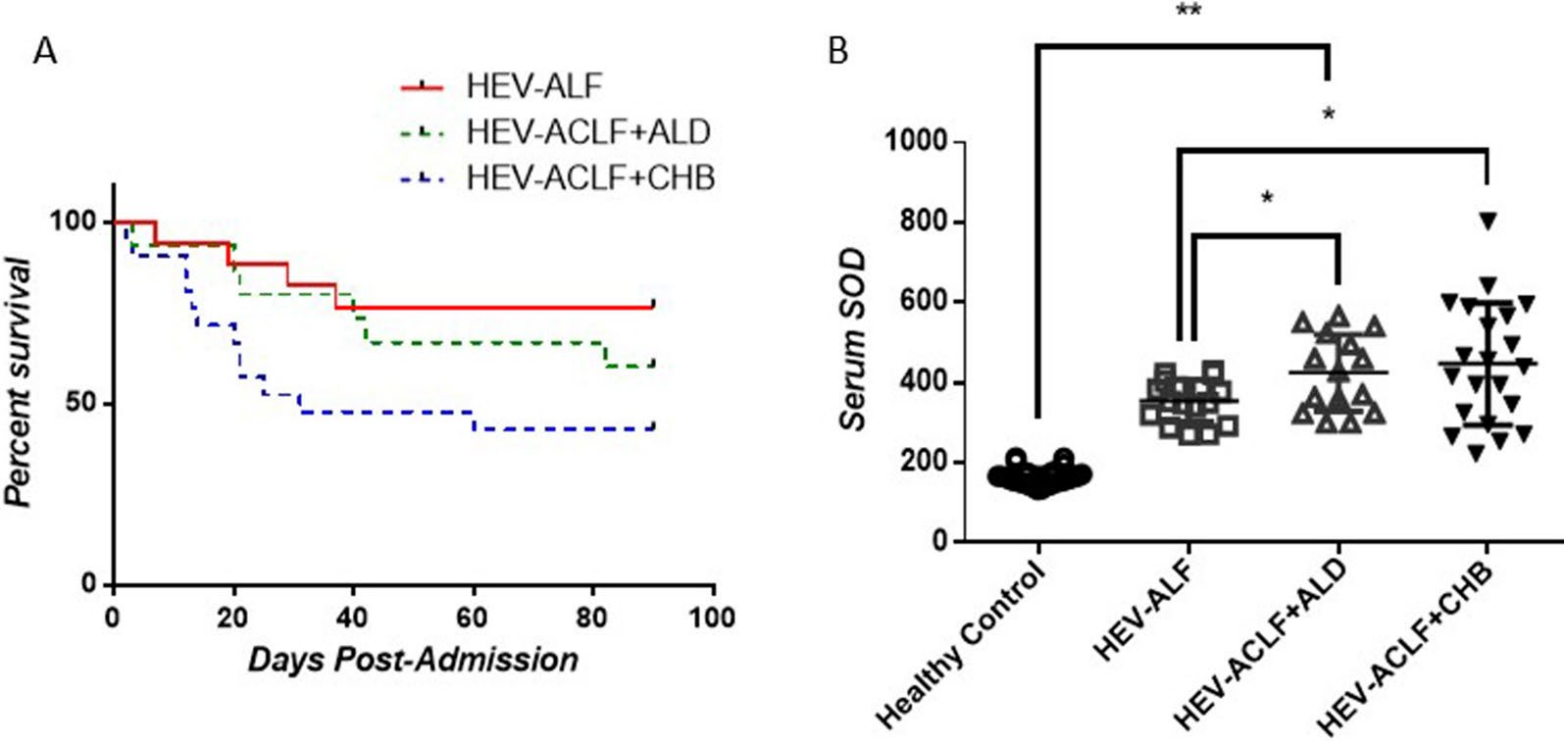
Kaplan–Meier analyses for survival and plasma SOD levels in HEV-induced liver failure patients. **A** HEV-ALF patients showed lower mortality rate than HEV-ACLF patients; **B** Plasma SOD levels were lower in HEV-ALF patients compared to HEV-ACLF patients. *P < 0.05, **P < 0.01

After infection, HEV usually causes acute symptoms and is one of the most common causes of liver failure worldwide. The mortality rate in the present study was 57.14% for HEV-ACLF patients on the background of CHB, and 40% for HEV-ACLF patients on the background of ALD; in contrast, patients with ALF caused by HEV showed a much lower mortality rate of 23.52%. Consistently, we found statistically significant differences in survival curves between HEV-ALF patients and HEV-ACLF on the background of CHB or ALD (Fig. 1A). There was a significantly lower plasma SOD level in HEV-ALF group compared with that in HEV-ACLF patients on the background of CHB or ALD (355.6 ± 12.72 U/mL vs 446.7 ± 33.11 U/mL, *P* < 0.05) & (355.6 ± 12.72 U/mL vs 424.4 ± 24.65 U/mL, *P* < 0.05). Moreover, the plasma SOD level was significantly higher in HEV-induced liver failure patients than in healthy controls (Fig. 1B).

### HEV-induced liver failure patients have high plasma SOD levels

**Fig. 2:**
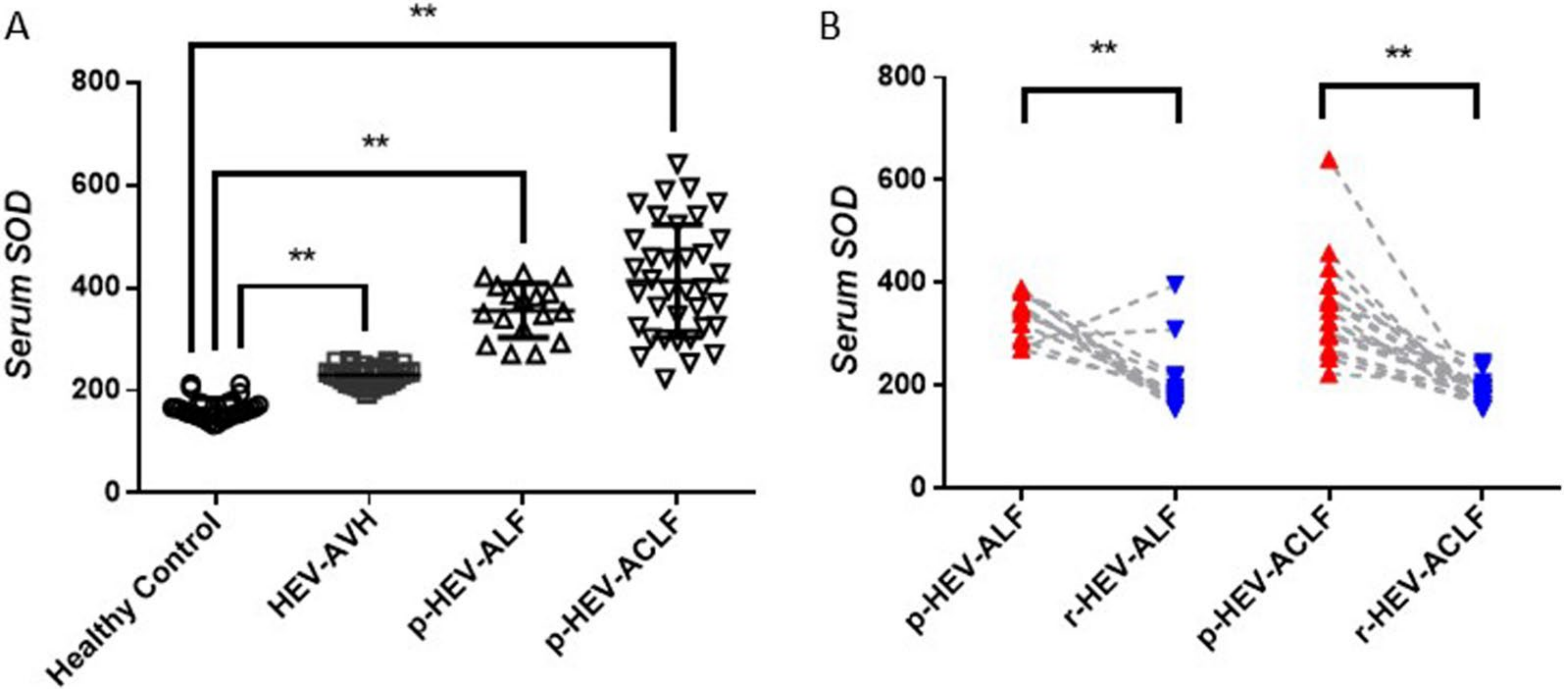
HEV-induced liver failure patients are associated with high plasma SOD levels. **A** SOD levels in progression stage of ALF (p-ALF) and ACLF (p-ACLF) were significantly higher compared with healthy controls or AVH patients; **B** In the remission stage of ALF (r-ALF) and ACLF (r-ACLF), SOD levels were decreased. **P < 0.01

We included another 30 HEV-AVH patients and compared plasma SOD levels between these AVH patients and heathy controls. As shown in Fig. 2A, significant differences were found (230.3 ± 3.29 U/mL vs 164.2 ± 3.82 U/mL, *P* < 0.05). We thus considered that the elevated plasma SOD level was associated with HEV infection and HEV-induced liver failure. We investigated plasma SOD level in all of the patients with HEV-induced liver failure and found a significantly higher SOD concentration in HEV-ALF patients compared with that in heathy controls (355.6 ± 12.72 U/mL vs 164.2 ± 3.82 U/mL, *P* < 0.01) and HEV-AVH patients (355.6 ± 12.72 U/mL vs 230.3 ± 3.29 U/mL, *P* < 0.01). Similar results were found in HEV-ACLF patients compared with that in healthy controls and HEV-AVH patients (437.4 ± 21.68 U/mL vs 164.2 ± 3.82 U/mL, *P* < 0.01) & (437.4 ± 21.68 U/mL vs 230.3 ± 3.29 U/mL, *P* < 0.01).

In the present study, we observed a decreased SOD level during remission stage compared with progression stage in 13 HEV-ALF and 18 HEV-ACLF surviving patients (338.6 ± 11.55 U/mL vs 206.5 ± 17.92 U/mL, *P* < 0.01) & (351.2 ± 22.55 U/mL vs 187.2 ± 5.75 U/mL, *P* < 0.01) (Fig. 2B).

### Plasma SOD levels are associated with mortality in patients with HEV-induced liver failure

**Fig. 3:**
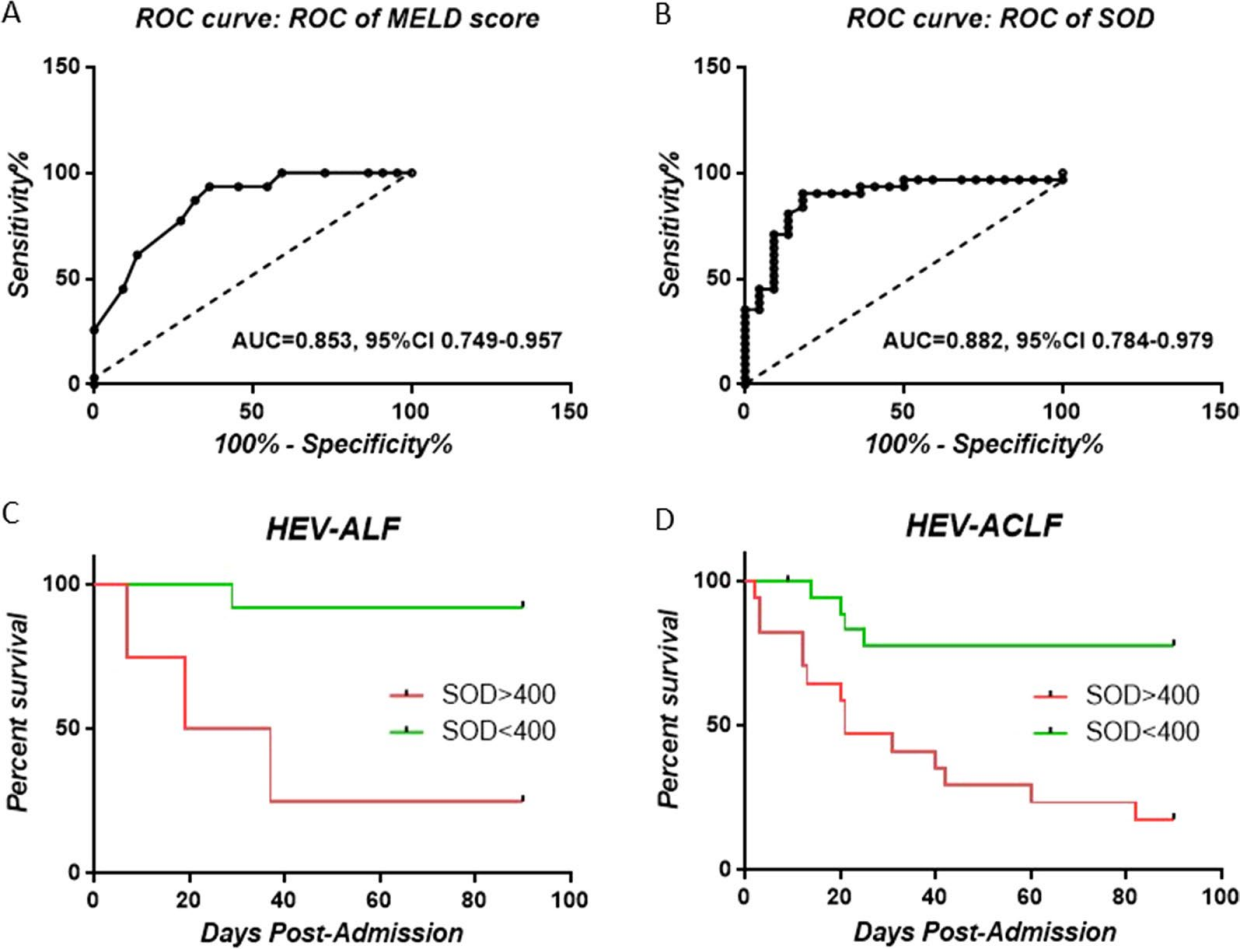
Kaplan–Meier analyses for survival according to plasma SOD levels. **A** ROC curve for MELD score; **B** ROC curve for plasma SOD; **C** Plasma SOD (above or below 400 U/mL) identifies HEV-ALF patients with higher mortality; **D** Plasma SOD (above or below 400 U/mL) identifies HEV-ACLF patients with higher mortality

In the current cohort, 22 out of the 53 patients died. Previously, we have found that MELD score > 25 was associated with high mortality risk in ACLF patients; here, we assessed the predictive value of MELD in HEV-induced liver failure patients. Using ROC methodology, we found 25.5 as the cutoff of MELD score (Fig. 3A). As plasma SOD level was significantly elevated in HEV-induced liver failure patients, we investigated whether it can be used for predicting disease outcomes. Using ROC methodology, the maximum sensitivity and specificity for SOD as a predictor of death within 90 days was 400 U/mL (Fig. 3B). The Kaplan–Meier analysis showed that patients with SOD values above this level had an increased risk of death (*P* < 0.01) (Fig. 3C, D).

### HEV-induced liver failure patients have high plasma HMGB1 levels

**Fig. 4:**
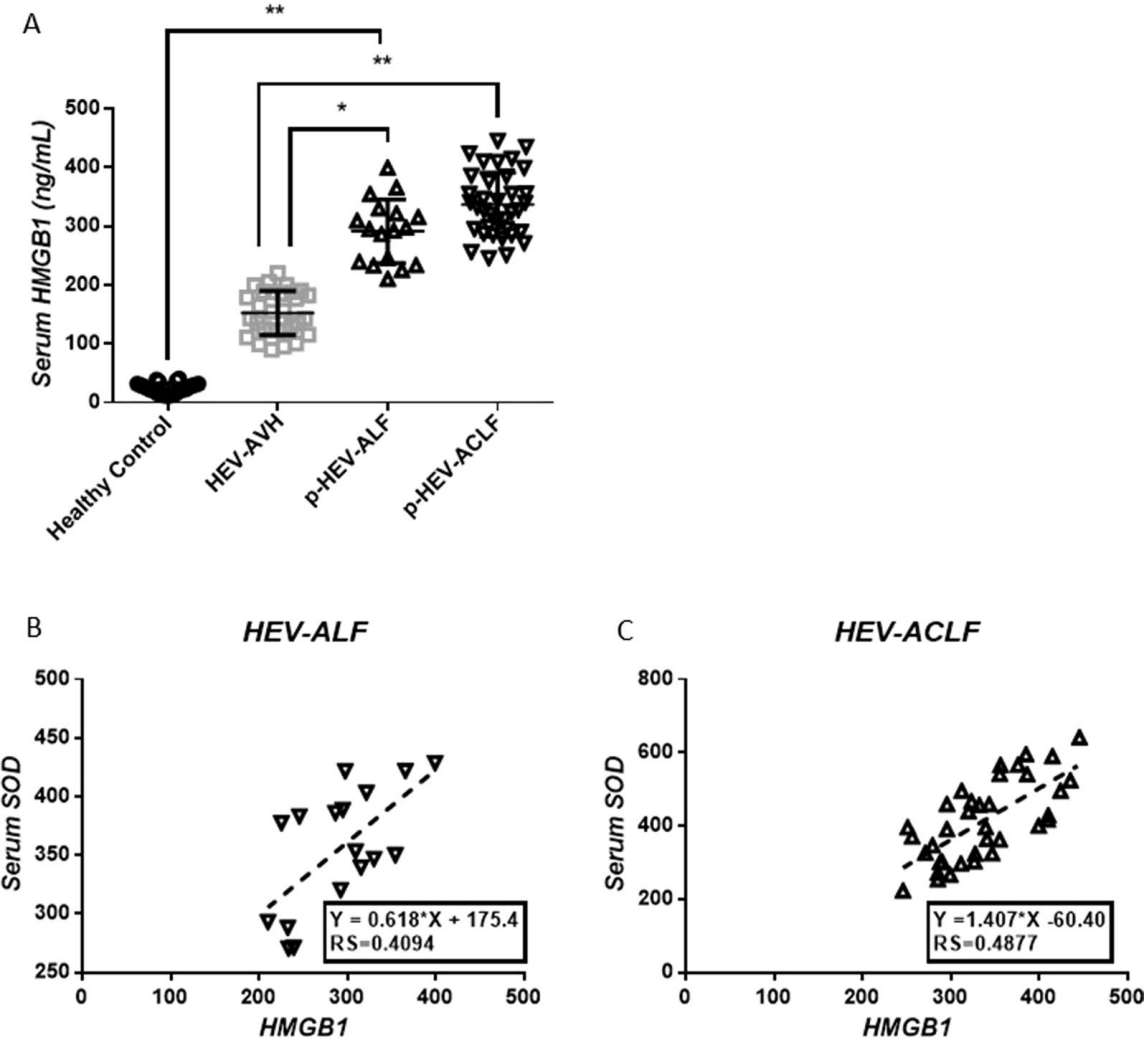
HEV-induced liver failure patients are associated with high plasma HMGB1 levels. **A** HMGB1 levels in progression stage of ALF (p-ALF) and ACLF (p-ACLF) were significantly increased compared with Healthy Controls or AVH patients; **B**, **C** HMGB1 levels were correlated with SOD levels. *P < 0.05, **P < 0.01

HMGB1, originally discovered as a nuclear binding protein, is involved in various hepatic diseases. A recently published study has shown elevated levels of circulating HMGB1 in HEV-AVH and HEV-ALF patients, and HMGB1 served as a predictor of disease severity in HEV-ALF patients. We investigated the plasma HMGB1 levels in patients with HEV-induced liver failure and found significantly higher levels in HEV-ALF patients compared with those in heathy controls (291.6 ± 13.16 ng/mL vs 23.9 ± 1.58 ng/mL, *P* < 0.01) and HEV-AVH patients (291.6 ± 13.16 ng/mL vs 152.3 ± 6.86 ng/mL, *P* < 0.01); similar results were found in HEV-ACLF patients (336.8 ± 9.08 ng/mL vs 23.9 ± 1.58 ng/mL, *P* < 0.01) & (336.8 ± 9.08 ng/mL vs 152.3 ± 6.86 ng/mL, *P* < 0.01) (Fig. 4A). Furthermore, a positive correlation was observed in both HEV-ALF and HEV-ACLF patients between the SOD level and the HMGB1 level (Fig. 4B, C).

### HEV leads to oxidative stress, HMGB1 release, and then apoptosis in hepatocytes

**Fig. 5:**
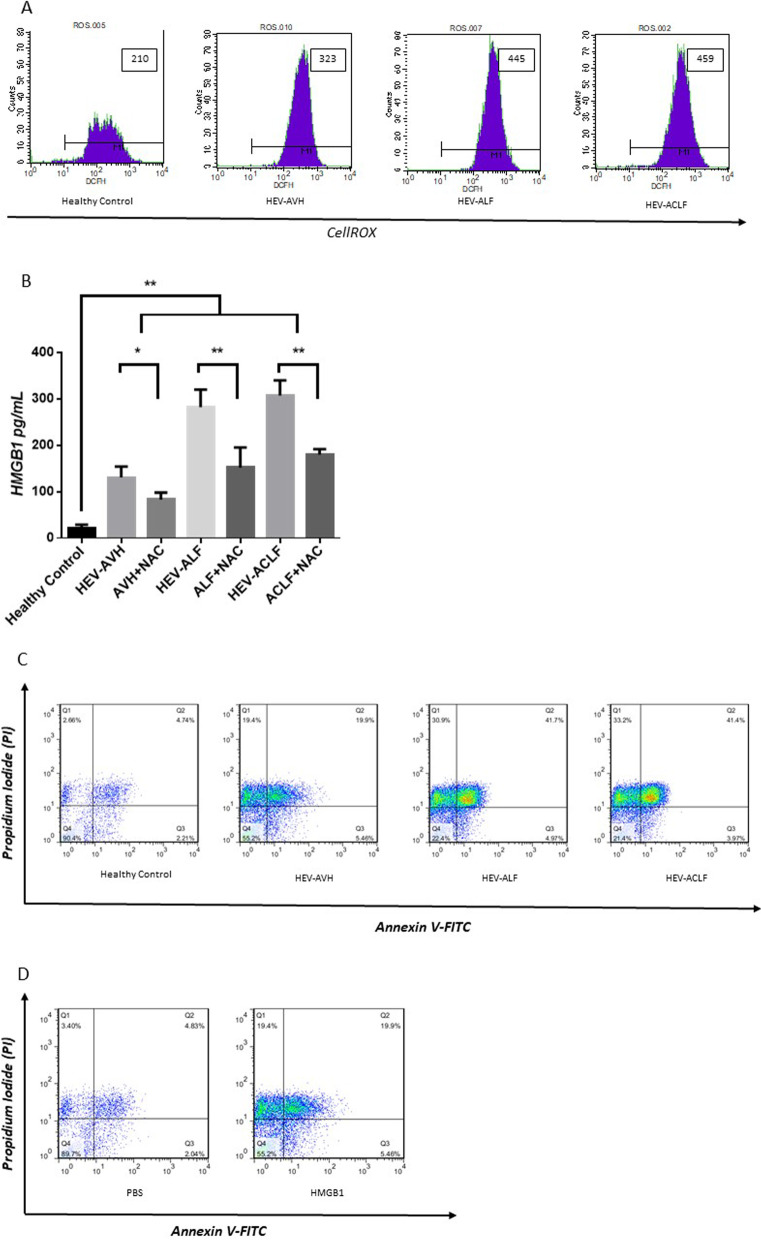
HEV promotes oxidative stress, HMGB1 release, and then apoptosis in hepatocytes. **A** Serum from HEV-induced liver diseases led to ROS accumulation in HL-7702 cells; **B** Serum from HEV-induced liver diseases led to HMGB1 secretion in HL-7702 cells, while inhibited by NAC; **C** Serum from HEV-induced liver diseases led to apoptosis in HL-7702 cells; **D** HMGB1 promoted apoptosis in HL-7702 cells. *P < 0.05, **P < 0.01

We first investigated the effect of HEV in hepatocytes. Specifically, we found that treatment with serum from HEV-AVH, HEV-ALF, and HEV-ACLF patients led to ROS accumulation and HMGB1 secretion in HL-7702 cells. Moreover, usage of N-Acetyl-L-cysteine (NAC), an antioxidant, successfully inhibited the HEV-induced HMGB1 secretion (Fig. 5A, B). To evaluate the proapoptotic effects of liver failure serum on HL-7702 cells, flow cytometry was used to determine the apoptotic rates. The results indicated that the apoptotic rates of the cells incubated with serum from HEV-AVH, HEV-ALF and HEV-ACLF patients were significantly higher than the apoptotic rates of the cells treated with serum from healthy controls (Fig. 5C). Incubation with HMGB1 led to apoptosis in HL-7702 cells (Fig. 5D).

## Discussion

Liver failure is a life-threatening disease with a high mortality rate. Despite high prevalence of HEV infection in developing countries [14], so far, few studies have focused on HEV-induced liver failure, and the disease pathophysiology is poorly understood.

In the present study, we found that patients with ACLF due to HEV infection on the background of CHB or ALD had higher peak laboratory abnormalities and poorer outcomes compared with those with HEV-ALF. The present study confirmed the previous finding that acute HEV infection can cause severe liver injury in patients with CHB and ALD. This indicates that chronic liver diseases, including CHB and ALD, have a negative impact on the clinical features of HEV infection, given that the mortality rates at 90 days were 57.14% in HEV-ACLF patients on the background of CHB and 40% in HEV-ACLF patients on the background of ALD, compared with 23.52% in patients with ALF caused by HEV. Although a high short-term mortality rate was found in patients with HEV-induced liver failure, so far, no effective prognostic biomarkers for evaluating the severity and prognosis of this disease have been identified. Thus, in the present study, we searched for a marker to reflect disease pathogenesis and predict the prognosis of HEV-induced liver failure.

Oxidative stress plays an important role in the pathogeneses of many human diseases, including ALF and ACLF. Here, we found a markedly increased circulating SOD levels in HEV-ALF/ACLF patients compared with those in healthy controls. Increased serum and liver tissue levels were found carbon tetrachloride-induced liver injury mice [15, 16]. As previous studies have revealed, ROS are crucial for NLRP3 inflammasome activation [17]. Injured/dead hepatocytes greatly increase oxidative stress during liver failure, which in turn contributes to further hepatocyte loss and impedes regeneration, thereby culminating in a vicious cycle during liver failure. Next, we assessed the prognostic value of plasma SOD level, which increases in an adaptive response to elevated systemic oxidative stress in HEV-induced liver failure. We found that patients with SOD level > 400 U/mL had significantly increased mortality.

We have previously found that the cytokine and chemokine levels vary during ALF and tend to recover during the remission stage [8, 12]. In the present study, we observed a decreased SOD level during the remission stage compared with the progression stage both in 13 HEV-ALF and in 18 HEV-ACLF patients, confirming the prognostic value of plasma SOD level in assessing the disease severity.

Moreover, we found that patients with HEV-ALF showed a significantly lower plasma SOD level than those with HEV-ACLF, which was consistent with the data of the disease mortality rate. We then investigated whether ongoing chronic liver diseases, including CHB and ALD, participate in elevated oxidative stress during HEV-ACLF. Liver failure is a disease characterized by oxidative stress, and it has been shown that mitochondria are the main source of ROS in hepatocytes when acutely and/or chronically exposed to a “damage” injury, including viruses, alcohol, environmental drugs, or therapeutical drugs [18]. A number of studies have linked HBV to the development of oxidative stress [19]. It is believed that HBV generates oxidative stress via altering mitochondrial function and modulating host gene expression, and the inhibition of HBV replication suppresses this ROS production [20]. To assess whether HEV also participates in ROS production, we collected another 30 HEV-AVH patients and found significant differences compared with healthy controls, indicating that HEV infection promotes oxidative stress during HEV-induced hepatitis and hepatic failure. Since oxidative stress plays a central role during ACLF, the increased oxidative stress during HEV-ACLF progression on the background of CHB or ALD led to a much higher oxidative stress together with a higher mortality rate compared with ALF simply caused by acute HEV infection.

We found that HEV led to ROS accumulation in HL-7702 cells. Published data on oxidative stress in HEV infection are very scarce. Some recent studies show that excessive oxidative stress caused by HEV infection is associated poor pregnancy outcomes [21, 22]. We think that HEV may serve as “damage” injury, promotes ROS production and then participates in pathogenesis of liver failure. The elevated ROS then promoted HMGB1 secretion, while the usage of an antioxidant (NAC) successfully inhibited this secretion. HMGB1 was originally discovered as a nuclear binding protein, and it has been implicated in various hepatic diseases [9, 23, 24]. The role of HMGB1 in promoting apoptosis has been confirmed in various diseases [25], high levels of HMGB1 in ALF patients/animals contributed to inflammatory response and multiple organ failure, serving as a bridge between hepatocyte injury and disease pathogenesis [26]. In the present study, serum from HEV-induced hepatic diseases, and HMGB1 promoted apoptosis in HL-7702 cells, which may participate in disease pathogenesis of HEV-induced hepatic diseases, including AVH, ALF and ACLF.

## Conclusion

Our results were derived from a large consortium over a 3-year long period, underscoring the generalizability of our findings; however, the limitations of the current study include those inherent to retrospective analysis, with potential biases such as selection bias. In conclusion, the present study revealed an increased level of SOD in patients with HEV-induced liver failure, which was mainly related to elevated oxidative stress during the disease pathogenesis. Early testing of plasma SOD may serve as a predictor of both HEV-ALF and HEV-ACLF outcomes. In addition, development of strategies to modulate oxidative stress might provide further insights into pathogenetic mechanisms, thereby providing a potential target for treating patients with HEV-induced liver failure.

## Data Availability

The datasets used and/or analyzed during the current study are available from the corresponding author on reasonable request.

## Abbreviations

HEV: Hepatitis E virus
AVH: Acute viral hepatitis
ALF: Acute liver failure
CHB: Chronic hepatitis B
ACLF: Acute-on-chronic liver failure
ALD: Alcoholic liver disease
HMGB1: High-mobility group box 1
ROS: Reactive oxygen species
SOD: Superoxide dismutase
TIPS: Trans-jugular intrahepatic portosystemic shunt
MELD: Model for end-stage liver disease
PCR: Polymerase chain reaction
NAC: N-Acetyl-L-cysteine
